# Tumor Budding in Upper Gastrointestinal Carcinomas: A Systematic Review and Meta-Analysis

**DOI:** 10.7759/cureus.70422

**Published:** 2024-09-29

**Authors:** Furat Almayouf

**Affiliations:** 1 Department of Laboratory and Blood Bank, Histopathology Section, Qassim Health Cluster, King Fahad Specialist Hospital, Buraydah, SAU

**Keywords:** gastrointestinal (gi) carcinomas, lymph node metastasis, lymphovascular invasion, prognosis and survival, tumor budding

## Abstract

Gastrointestinal (GI) carcinomas represent a heterogeneous composition of malignancies that stem from the organs of the GI tract. They are among the most prevalent and are associated with high mortality alongside morbidity rates. This study utilized the guidelines set forth by Preferred Reporting Items for Systematic Reviews and Meta-Analyses (PRISMA), whereby four medical databases were searched for relevant scholarly publications published between 2010 and 2024. These databases were PubMed, Web of Science, and ScienceDirect. Risk of bias (RoB) for cohort, case-controlled, and cross-sectional studies was assessed using the Newcastle-Ottawa Scale (NOS), whereas randomized controlled trials (RCTs) used the Cochrane (RoB) tool. With the assumption that the observed estimate of the treatment effect differs between the included studies, a random effect meta-analysis was carried out. In the meta-analysis, fifteen trials with 7607 patients were considered. The findings indicate a substantial correlation between high-grade tumor budding and tumor stage, (χ²=480472.97, P<0.00001) with a mean difference of −6.18 at 95% confidence interval (95% CI) (−14.66 to 2.30), tumor differentiation (χ²=23.31, P<0.00001) with a mean difference of −12.60 at 95% CI (−35.89 to 10.68), lymph vascular invasion (χ²=29.59, P<0.00001) with a mean difference of −5.03 at 95% CI (−11.26 to 1.21), and lymph node metastasis (χ²​​​​​​​=158.30, P<0.00001) with a mean difference of −3.44 at 95% CI (−4.72, −1.78). Furthermore, in upper gastrointestinal (UGI) patients, high-grade tumor budding was associated with a negative five-year overall survival (P<0.00001) and a mean difference of −0.09 at 95% CI (−0.20 to 0.02). In regards to the risk of bias, most of the retrospective, prospective, case-control, and cohort studies 10/14 were of satisfactory quality. Moreover, 5/7 of the clinical trials had a low risk of bias. However, the funnel plot indicated that there is a probability of publication bias in favor of tumor budding. The study revealed a significant link between tumor budding and key prognostic factors-overall survival, lymph node metastasis, tumor differentiation, and lymphovascular invasion-in upper gastrointestinal carcinomas. High-grade tumor budding is associated with poor clinicopathological characteristics and a five-year overall survival. Tumor budding may serve as a unique prognostic marker. To confirm these results, further research with larger preoperative UGI biopsies is recommended.

## Introduction and background

Gastrointestinal (GI) carcinomas represent a heterogeneous composition of malignancies that stem from the organs of the GI tract. They are among the most prevalent and are associated with high mortality alongside morbidity rates and represent more than 25% of all cancer cases [1]. GI carcinomas had an age-standardized incidence rate (ASIR) of 61.9 per 100,000 person-years at a 95% confidence interval (CI) of 56.1−67.6 [1]. As to the Global Burden of Diseases, Injuries, and Risk Factors Study (GBD) 2017, 36.2% of fatalities associated with neoplasms were attributable to gastrointestinal malignancies [2]. Research shows that GI carcinomas-related incidence and mortality were, in absolute numbers, about five million and three million, respectively [3].

Upper gastrointestinal (UGI) carcinomas affect the upper sections of the GI tract and comprise esophagogastric, biliary, liver, and pancreatic tumor types [4]. UGI tumors are the second most prevalent cause of mortality among all GI cancers. While the global burden of upper GI carcinomas remains high, it varies widely across regions; East Asia and Sub-Saharan Africa had the highest incidence and mortality rates, with the lowest rates observed in Central America and West Africa [5]. UGI cancer incidence and mortality rates are higher among men than women [6].

Diagnostic technologies have greatly improved, but despite the effort to diagnose and treat GI carcinomas, the prognosis for patients is still wanting [7]. Imai was the pioneer in identifying tumor budding (TB) in the 1950s [8], describing it as a "sprouting" phenomenon at the invasive edge of cancer cells. While it is widely recognized as a negative prognostic indicator for colorectal carcinoma, its significance is increasingly being recognized in upper gastrointestinal (UGI) carcinoma as well. Hase et al. also demonstrated cellular activity in 1993, tantamount to TB, where cells depicted reduced survival with an increase in budding [8]. In colorectal cancers, tumor buds show a lack of cell polarity that could lead to fibroblastoid morphology, downregulation of the adhesion protein E-cadherin, and nuclear translocation of β-catenin [7,9].

Unlike in colorectal cancer, where the International Tumor Budding Consensus Conference (ITBCC) has established standardized guidelines, there is no universally accepted grading criterion for TB in UGI cancers. The ITBCC classifies TB in colorectal carcinoma into low-grade (0-4 buds), intermediate (5-9 buds), and high-grade (≥10 buds) based on hematoxylin and eosin staining at a magnification of 200×, normalized to a 0.785 mm² field [8].

While these guidelines provide a standardized, structured approach for TB classification in colorectal cancers, their applicability in UGI cancers remains inconsistent and an ongoing debate. Berg et al. discussed the criterion for grading UGI TB based on the ITBCC colorectal carcinoma TB reporting criteria, using hematoxylin and eosin staining, but noted the need for further validation tests in the context of UGI cancers [8]. Ueono et al., on the other hand, used cytokeratin staining and a different magnification (250×), averaging bud counts over 10 high-power fields (hpfs) on pancreatic ductal adenocarcinoma [9].

A comparison of the above two studies highlights the variability in methodologies across studies. Other studies examined multiple fields and similarly adopted diverse approaches and established varying average bud counts across 5-10 hpf [10]. Given this variability, our study adopts the ITBCC grading guidelines from colorectal cancer as a reference point, all while acknowledging the ongoing need for standardized criteria specifically tailored to UGI carcinomas. This review aims to synthesize current evidence on the prognostic significance of TB and desmoplastic reaction in UGI carcinoma.

## Review

Methodology

This study utilized the guidelines set forth by Preferred Reporting Items for Systematic Reviews and Meta-Analyses (PRISMA), through which four medical databases were searched for relevant scholarly publications published between 2010 and 2024. These databases were PubMed, Web of Science, ScienceDirect, and Google Scholar.

Keywords

A combination of keywords was used in searching for relevant articles for review, general terms included: “Upper Gastrointestinal Carcinoma”/“Esophageal Cancer”/“Gastric Cancer”/“Gastrointestinal Cancer”. Specific terms related to the pathology of interest include: “Tumor Budding”/“Desmoplastic Reaction”. Prognostic and clinical outcome terms included: “Prognosis”/“Survival”/“Histopathology”/“Patient Outcomes”/“Disease Progression”.

Search Strategy

The databases searched were PubMed, Web of Science, and ScienceDirect. Specific keywords and boolean operators were tailored for each database to enhance search precision. For PubMed, we utilized Medical Subject Headings (MeSHs) terms such as "Upper Gastrointestinal Carcinoma" AND "Tumor Budding" OR "Desmoplastic Reaction" AND "Prognosis" OR "Survival." Web of Science required the use of similar terms with additional filters applied, including date range (2010-2024) and study type filters (clinical trials, cohort studies, etc.). ScienceDirect was searched using keywords with a focus on "Upper Gastrointestinal Cancer" and "Histopathology," with boolean combinations of “AND” and “OR” to link prognostic factors like "Survival Rates" or "Tumor Differentiation." Only peer-reviewed articles were considered, and the search was restricted to English-language publications.

Eligibility, data extraction, and management

A predetermined inclusion-exclusion criterion was utilized to rigorously scrutinize the retrieved studies for eligibility.

Inclusion Criteria

Studies published between 2010 and 2024. Only studies published in the English language were included. We included randomized controlled trials, cohort studies, observational studies, retrospective studies, and prospective observational studies; studies that focused on humans with upper gastrointestinal carcinomas like esophageal cancer, gastric cancer, and intestinal-type gastric adenocarcinoma. Our focus was on studies that investigated the prognostic importance of tumor budding and desmoplastic reaction in upper gastrointestinal carcinomas. We were interested in studies investigating the prognosis, survival rates, histopathology findings, and overall patient outcomes.

Exclusion Criteria

Non-human studies, in-vitro studies. Non-relevant pathology studies especially those focusing on colorectal, rectal, and other cancers of the lower gastrointestinal tract. Systematic reviews, meta-analyses, and studies that did not focus on patients with upper gastrointestinal carcinomas were excluded. Studies that did not assess tumor budding, desmoplastic reaction, or clinical outcomes relevant to upper gastrointestinal carcinomas were excluded to ensure relevance and specificity to the review objectives.

The above inclusion-exclusion criteria were used to ensure that only high-quality, full-text, relevant articles were included. We ensured that the inclusion was geared towards offering meaningful information on the prognosis of tumor budding and desmoplastic reactions in patients suffering from carcinomas of the upper gastrointestinal tract. Among others, we focused on esophageal cancer, gastric cancer, and intestinal-type gastric adenocarcinoma. Further, the study adopted the three TB grouping suggested by tumor budding score recommendations for colorectal cancer from the International Tumor Budding Consensus Conference (ITBCC): BD1 (0-4 buds/0.785 mm^2^), BD2 (5-9 buds/0.785 mm^2^), and BD3 (10 or more buds/0.785 mm^2^).

Statistical analysis

Relevant metrics to aid in our meta-analysis were determined and computed-risk ratio (RR), standard error (SE), random effect (RE), and confidence interval (CI), were computed using the Resource Manager (RevMan) version 5.4.1 (The Cochrane Collaboration, London, UK). Heterogeneity in the included studies was computed via a multivariate random-effects regression analysis, whose I^2^ values were consequently classified into low, low-to-moderate, moderate-to-high, and high heterogeneity. We used funnel plot asymmetry tests and summary bar graphs to determine bias in each publication and specific items therein. A threshold (p=0.05) was used to determine statistical significance.

Results

**Figure 1:**
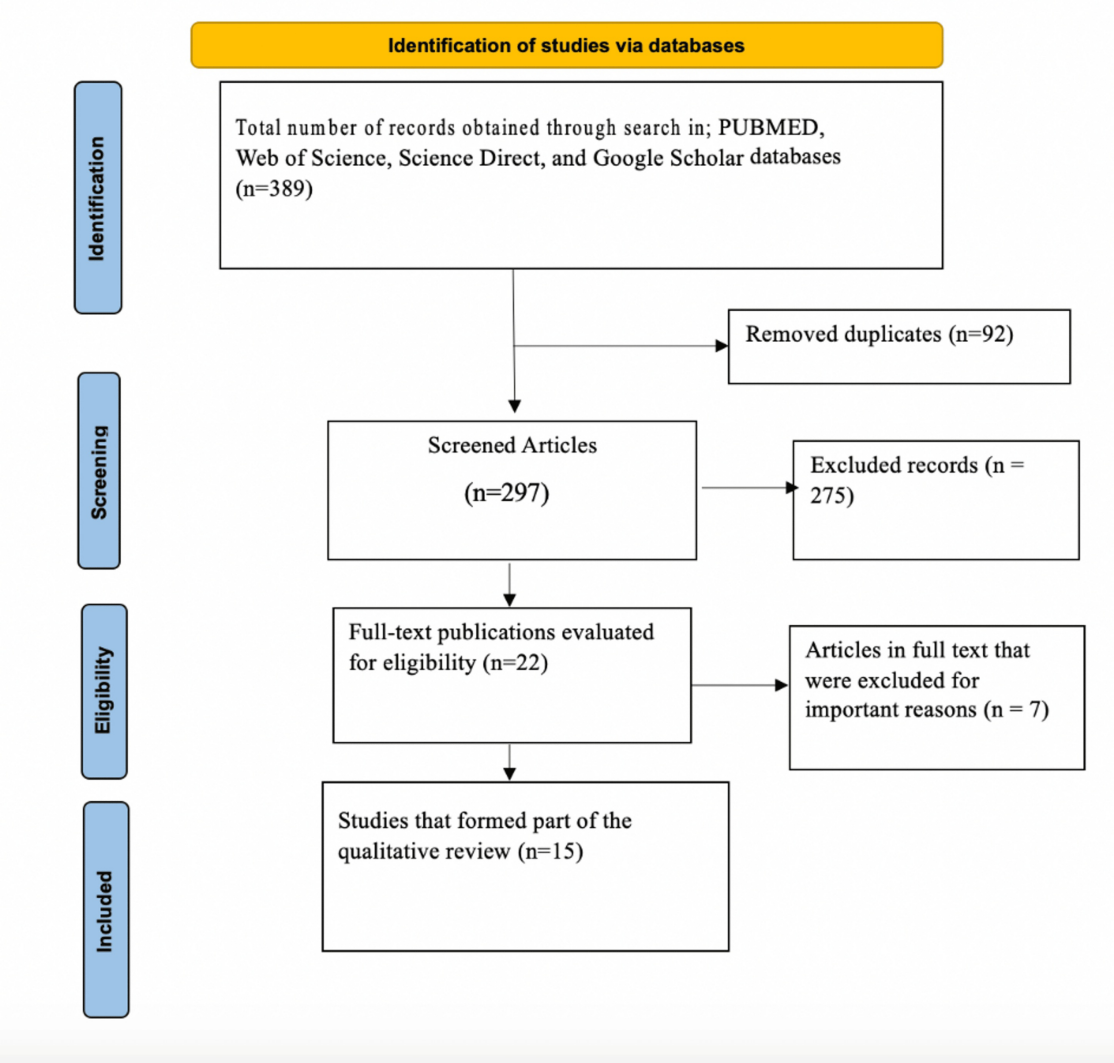
PRISMA flow diagram. PRISMA: Preferred Reporting Items for Systematic Reviews and Meta-Analyses.

A search of the electronic database produced 389 articles. Based on their titles and abstracts, 275 of them were not eligible, and 92 of these were duplicates. After reviewing the entire texts of the remaining 22 articles, seven more were eliminated due to a lack of sufficient data and failure to address the outcome of interest. In the end, 15 studies were found to be eligible and were added to the systematic review. The PRISMA 2020 flow diagram shows the selection procedure (Figure 1).

Qualitative synthesis

Table 1 shows the articles that were part of the review; every article examined tumor budding in cases of upper gastrointestinal carcinomas. The research was carried out in different parts of the world and published in English. The included studies were carried out in various regions/countries; eight of the studies came from the United States, while one each from Singapore, England, Germany, Turkey, Japan, Vietnam, and the Republic of Korea, respectively. The included studies had a total sample size of 7607 people, with the study with the lowest sample size having 42 participants and the study with the largest sample size having 4660 participants. The intervention used in all the investigations was tumor budding.

**Table 1:** Characteristics of the studies. LVI: lymphovascular invasion, DR: desmoplastic reaction, GSCC: gastric squamous cell carcinoma, GAC: gastric adenocarcinoma, ESGE: European Society of Gastroenterology, LNM: metastases to lymph nodes, SESCC: surface esophageal squamous cell carcinoma, OS: overall health and survival, ER: endoscopic resection, ESD: endoscopic resection submucosal dissection, GC: gastric cancer, HER: electronic health records, SCC: squamous cell carcinoma, ITBCC: The International Tumor Budding Consensus Conference, TILs: tumor-infiltrating lymphocytes, VIR: vascular, CC-IR: insulin receptor on cancer cells, RFS: relapse-free survival.

Authors	Region	Study design	Sample	Intervention	Inclusion	Results/conclusion
Li et al. 2023 [11]	Singapore	A retrospective study	150	Tumor budding	Individuals who underwent radical esophagectomy with dissection of lymph nodes between 1990 and 2004 and had primary ESCC at the pT1b stage.	Low pTIL level, lower tumor location, and lymphovascular invasion (LVI) are associated with unfavorable prognosis in pT1b ESCC patients. A nomogram based on these factors shows good discrimination, facilitating personalized survival prediction.
Beer et al. 2022 [12]	United States	A retrospective study	78	Tumor budding	To meet the inclusion criteria, an invasive tumor's preoperative biopsy and its resection specimen, both kept at the Department of Pathology, were required.	In preoperative biopsies, budding foci may help to increase the predictive precision for esophageal carcinomas.
Yavuz et al. 2024 [13]	United States	A retrospective study	130	Tumor budding	Individuals treated for gastric cancer between 2004 and 2019 who had a whole or partial gastrectomy.	In GAC, TB might be a useful prognostic indicator. To clarify its place in pathology reporting methods and the predictive power of DR and TILs, more research is necessary.
Dhingra et al. 2021 [14]	England	A retrospective study	42	Tumor budding	Individuals whose final pathologic evaluation ESD material revealed BE with invasive adenocarcinoma.	Tumor budding frequently correlates with other high-risk characteristics, requiring endoscopic correlation for therapy, according to the study, which validates ESGE guidelines for high-risk pathologic findings in esophageal adenocarcinoma.
Yu et al. 2024 [15]	United States	A retrospective study	474	Tumor budding	Patients with SESCC who had esophagectomy between January 1, 2009, and January 31, 2016.	Tumor outgrowth has been used more often in SESCC pathology diagnosis and standard reporting because the study demonstrated that it is an independent risk factor for LNM.
Wang et al. 2023 [16]	United States	Clinical controlled trial	4660	Tumor budding	Patients with esophageal cancer and esophagectomy were included in the study; pT1 stage tumors, squamous cell carcinoma, fewer lymph nodes, prior malignancies, insufficient clinical data, and low- or high-grade neoplasia were among the exclusion criteria.	In order to help with future decision-making regarding the treatment of patients undergoing endoscopic resection, we have created a practical nomogram model that predicts LNM risk for patients with superficial ESCC.
Landau et al. 2014 [17]	United States	A retrospective study	210	Tumor budding	Representative tumor slides from patients treated with esophagectomy without induction therapy for stage pT1 esophageal or gastroesophageal junction adenocarcinoma at the University of Pittsburgh Medical Center between 1996 and 2013 were available for examination.	It is important to include tumor budding in thorough pathologic risk evaluations because it is a poor prognostic marker for superficial esophageal adenocarcinoma, regardless of nodal metastasis.
Davison et al. 2016 [18]	United States	Cohort study	210	Tumor budding	individuals who underwent esophagectomy between 1996 and 2012 and had T1 EAC	The pathologic features of primary superficial EACs can be used to categorize patients into high-risk or low-risk groups according on whether they require adjuvant therapy or endoscopic resection or induction.
Dao et al. 2020 [19]	Vietnam	A retrospective study	109	Tumor budding	Patients treated between 2012 and 2015 for stomach cancer, ranging in age from 28 to 80. The only patients chosen had malignancies that were not yet treated.	According to the study, patients with gastric cancer who have different Bd grades can be successfully categorized for therapy and prognosis using the ITBCC criteria.
Kemi et al. 2019 [20]	United States	Retrospective cohort study	583	Tumor budding	Patients with stomach adenocarcinomas who had surgery between 1983 and 2016	When a patient has considerable tumor budding, the five-year survival rate for patients with intestinal-type adenocarcinomas is lower than that of diffuse-type adenocarcinomas. In cases of gastric adenocarcinoma, high tumor budding is an independent prognostic indicator.
Teramoto et al. 2013 [21]	Japan		79	Tumor budding	Patients diagnosed with esophageal SCC had esophagectomy procedures performed between May 1988 and July 2005.	Tumor budding, which indicates tumor activity and may be a prognostic indication in early-stage esophageal SCC, indicates that esophagectomy has a good three-year survival rate.
Baydas et al. 2023 [22]	Turkey	A retrospective, cross-sectional study	104	Tumor budding	Individuals who underwent surgery and received a diagnosis of stomach cancer between 2012 and 2020	The study found that patients aged 60 or older tended to have a lower body mass index, a higher N stage, lymphovascular invasion, tumor recurrence, increased tumor budding, and a longer average time from diagnosis to the last follow-up.
Lee et al. 2023 [23]	Republic of Korea	Case controlled study	235	Tumor budding	All EGC patients who underwent surgery between January 2010 and October 2023, regardless of prior ER status.	The discovery of multiple tumor budding (TB) foci near the invasive front highlighted significant concerns about lymph node metastasis (LNM). Consequently, the study suggests that tumor budding could play a crucial role in the development of LNM.
Heckl et al. 2019 [24]	Germany	Prospective study	467	Tumor budding	Every patient who between 1997 and 2009 had a whole or partial gastrectomy for esophageal-gastric junction or stomach cancer.	The biological significance and frequent expression of VIR and CC-IR in GC, along with their correlation with HER2 status, provide new opportunities for potential therapeutic approaches in GC.
Pun et al. 2023 [25]	United States	A retrospective study	76	Tumor budding	Individuals who underwent primary gastrectomy for intestinal-type gastric cancer between 2000 and 2018	Pathologic risk factors are linked to both tumor budding and desmoplastic reaction; whereas the desmoplastic response is associated with RFS, tumor budding predicts OS and RFS.

Table 2 summarizes findings from several studies that evaluate high-grade tumor budding (TB) and its clinicopathological characteristics. These studies, conducted in various settings, included sample sizes ranging from 28 to 109 and used different magnifications for high-power fields (HPF) and areas of the field (mm²). The criteria for high-grade TB evaluation varied, with thresholds such as >10 TB/HPF and ≥5 TB/HPF, indicating a lack of standardized thresholds across different research settings. Several studies, including the one by Ulase et al., applied the ITBCC criteria, originally established for colorectal cancer, to assess tumor budding in gastric cancer patients (Table 2). Tumor buds were defined as clusters of less than five cells within a selected "hotspot" after reviewing all available invasive tumor slides. The total number of buds should be reported in an area measuring 0.785 mm², which corresponds to a 20× field in some microscopes. Tumor budding was classified into three tiers: low (Bd1) with 0-4 buds, intermediate (Bd2) with 5-9 buds, and high (Bd3) with more than 10 buds. Most studies focused on invasive adenocarcinoma and intestinal-type gastric cancer, reflecting their prevalence in TB research.

**Table 2:** Criteria and grading systems for tumor budding assessment in gastric cancer across different studies. H&E: hematoxylin and eosin; IHC: immunohistochemistry, HPF: high power field.

Authors	Year	Straining	Definition of tumor budding	Area of field	Power of objectives	High-grade TB	Low-grade TB
Li et al. [11]	2023	H&E- Pancytokeratin	1 to <5	0.955	200×	≥16 TBs per 20× objective lens	0-4 TBs per 20× objective lens
Beer et al. [12]	2022	H&E, IHC	1 to ≤4	N/R	20×	≥16 TBs per 20× objective	0-4 TBs per 20× objective
Yavuz et al. [13]	2024	H&E	1 to ≤4	0.785	200×	Grade 3 (>10 TB) per 0.785 mm²	Grade 1 (0-4 TB) per 0.785 mm²
Dhingra et al. [14]	2021	H&E	1 to <5	0.785	200×	>10 TBs per HPF	≤10 TBs per HPF
Yu [15]	2024	H&E, IHC	1 to ≤4	0.785	20×	≥5 Budding foci per 20× objective lens	1-4 Budding foci per 20× objective lens
Wang et al. [16]	2023	H&E	1 to ≤4	N/R	20×	≥5 Budding foci	1-4 Budding foci
Landau et al. [17]	2014	H&E, IHC	1 to <5	0.785	200×	Extensive (≥3 fields with >5 buds per field)	None or focal (0–2 fields with ≤5 buds per field)
Davison et al. [18]	2016	H&E	<50% Tubular, papillary, or gland forming; poorly differentiated	0.785	200×	≥5 Buds per HPF	<5 Buds per HPF
Dao et al. [19]	2020	H&E	1 to ≤4	0.785	20×	≥10 Buds per HPF	<10 Buds per HPF
Kemi et al. [20]	2019	H&E	1 to ≤ 5	0.785	200×	≥10 Buds per HPF	<10 Buds per HPF
Teramoto et al. [21]	2013	H&E	Frequent (≥3 budding foci per 20× objective lens)	N/R	20x	≥3 Buds per HPF	<3 Buds per HPF
Baydas et al. [22]	2023	H&E	1 to ≤ 5	0.785	200×	≥10 Buds per HPF	<10 Buds per HPF
Lee et al. [23]	2023	H&E IHC	1 to <5	0.785	200×	High-grade TB (>5 buds per HPF)	Low-grade TB (≤5 buds per HPF)
Heckl et al. [24]	2019	H&E	1 to <5	0.785	20×	Bd3: ≥10	Bd1/Bd2: <10 buds
Pun et al. [25]	2023	HE, IHC	1 to <5	0.785	200×	>10 Buds per HPF	≤10 Buds per HPF

Quantitative synthesis

**Figure 2:**
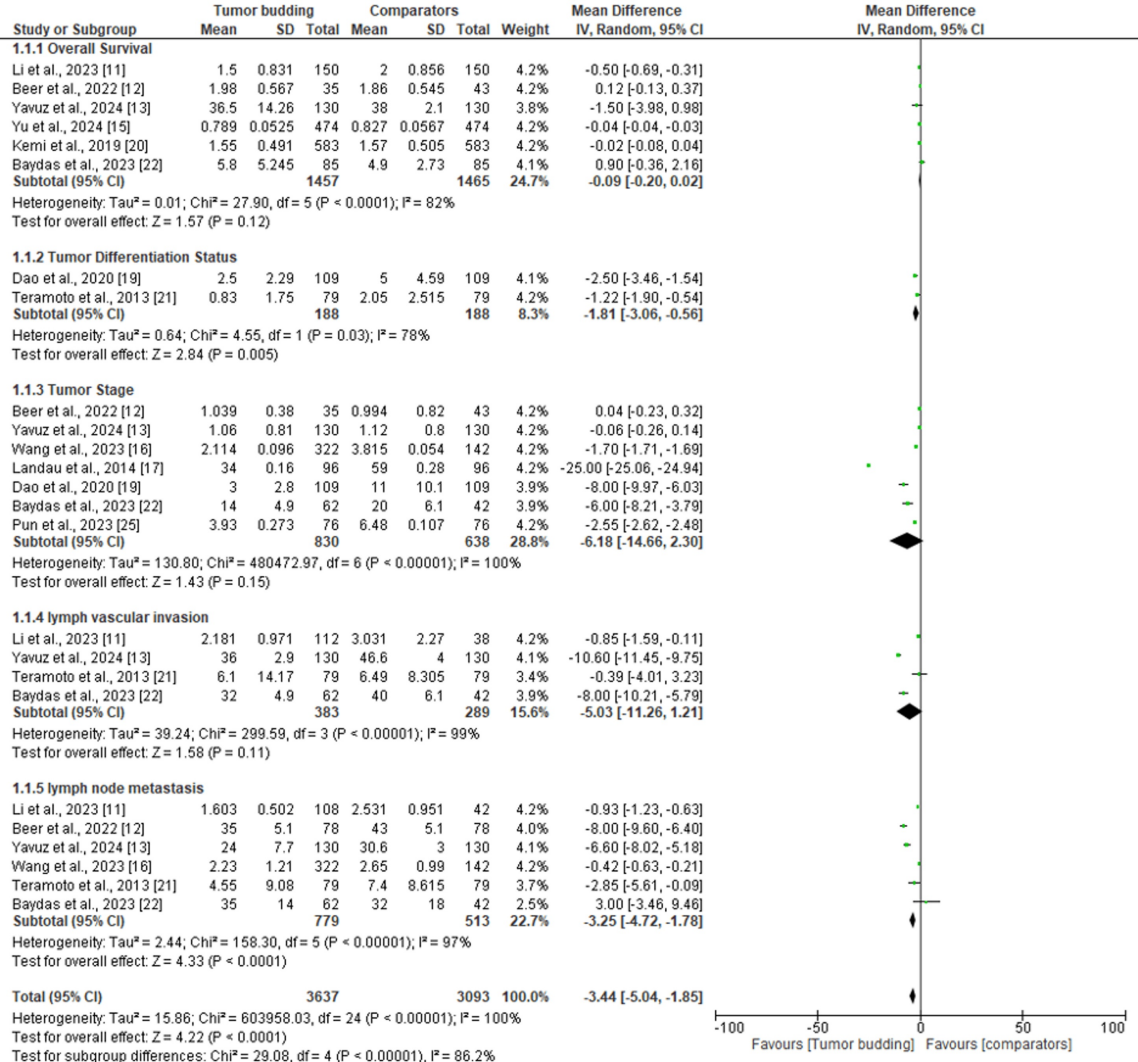
Forest plot of the association between tumor budding and gastrointestinal carcinoma factors. Note: This image is the author's own creation.

Figure 2 shows the relationship between overall survival, UGC lymph node metastasis, tumor differentiation status, depth of tumor stage, and lymph vascular penetration. The results show that there is a meaningful association between tumor budding and overall survival (P<0.00001), with a mean difference of -0.09 at 95% CI (-0.20 to 0.02). The test of heterogeneity of this factor was also significant (Τ^2^=0.01, I^2^=82%). In regards to tumor differentiation status, the results show that there is a statistically significant association between tumor budding and tumor differentiation status (χ²=23.31, P<0.00001) with a mean difference of -12.60 at 95% CI (-35.89 to 10.68). There was also a high heterogeneity of the studies analyzed (I^2^=78%).

Depth of the tumor stage was also shown to have a statistically significant association with tumor budding (χ²=480472.97, P<0.00001) with MD=-6.18 at 95% CI (-14.66 to 2.30). There was also a high heterogeneity of the included studies (I^2^=100%). There was a significant relationship between lymph vascular invasion and tumor budding (χ²=29.59, P<0.00001) with a mean difference of -5.03 at 95% CI (-11.26 to 1.21). The heterogeneity of the studies analyzed was also very high (I^2^=99%). Moreover, there was a statistically significant association between lymph node metastasis and tumor budding (χ²=158.30, P<0.00001) with a mean difference of -3.44 at 95% CI (-4.72, -1.78). The studies included in the analysis of this factor were also shown to have a high heterogeneity (I^2^=97%).

Risk of bias based on quality assessment of cross-sectional studies-Newcastle-Ottawa Quality Assessment Scale (NOS)

The Newcastle-Ottawa Quality Assessment Scale (NOS) was utilized to evaluate the quality of the Cohort, case-control, and retrospective/prospective studies, as presented in Table 3. It was noted that ten of the fourteen studies that were reviewed [11-13,15,17,19-22,25] were of satisfactory quality. At the same time, four of the fourteen studies [14,18,23,24] were of moderate quality.

**Table 3:** Risk of bias based on quality assessment of cohort, case-control, and retrospective/prospective studies-NOS. Selection: Q1. Is the exposure cohort representative?, *Q2. How was the non-exposure cohort chosen?, *Q3. Determination of the exposure?, *Q4. Proof that the desired outcome did not exist at the outset of the research?, **Comparability Q5. Is the cohort comparable based on the analysis or design?, **Outcome: Q6: Evaluation of the result?, *Q7: Test Statistics? **indicate satisfactory for selection and outcome; **indicate satisfactory for comparability.

Authors	Selection				Comparability	Outcome	
	The exposed cohort's representativeness	The non-exposed cohort's selection	Determining the exposure	Non-participants	Comparability of cohorts based on the analysis or design	Evaluation of the result	Test of statistics
Li et al. [11]	*	*	*	?	**	*	*
Beer et al. [12]	*	*	*	?	**	*	*
Yavuz et al. [13]	*	*	*	?	**	*	*
Dhingra et al. [14]	*	*	*	?	*	*	*
Yu [15]	*	*	*	?	**	*	*
Landau et al. [17]	*	*	*	?	**	*	*
Davison et al. [18]	*	*	*	?	*	*	*
Dao et al. [19]	*	*	*	?	**	*	*
Kemi et al. [20]	*	*	*	?	**	*	*
Teramoto et al. [21]	*	*	*	?	**	*	*
Baydas et al. [22]	*	*	*	?	*	*	*
Lee et al. [23]	*	*	*	?	*	*	*
Heckl et al. [24]	*	*	*	?	**	*	*
Pun et al. [25]	*	*	*	?	**	*	*

Risk of bias based on quality assessment of clinical control trial-Cochrane risk of bias tool

**Figure 3:**
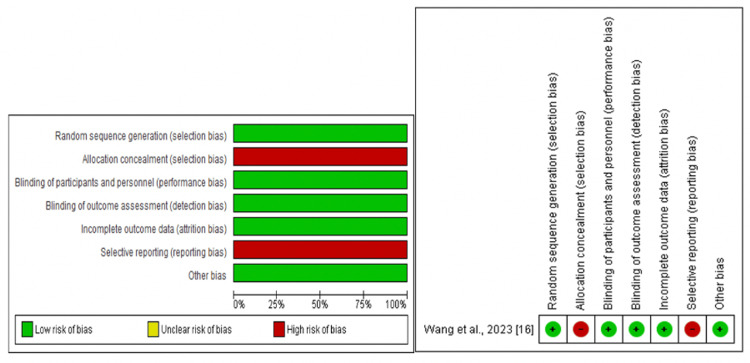
Summary of risk of bias for the included clinical trial. Note: This image is the author's own creation.

The results shown in Figure 3 indicate that the clinical trial included had a low risk of bias in most of the items 5/7. However, 2/7 of the items had a high risk of bias (allocation concealment and selective reporting).

**Figure 4:**
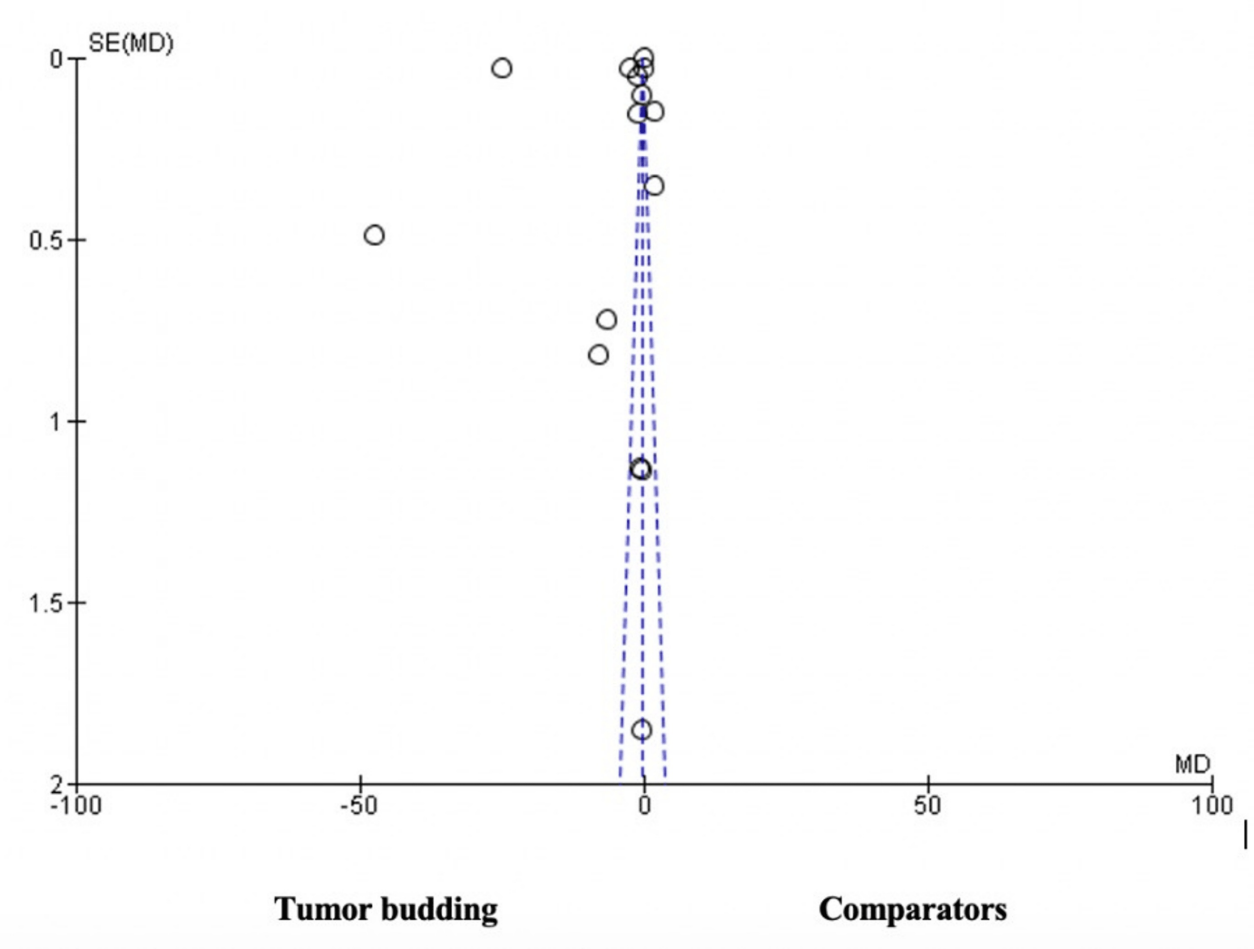
Funnel plot of the publication bias. SE: standard error, MD: means difference. Note: This image is the author's own creation.

As shown in Figure 4, the funnel plot depicts an asymmetrical funnel, with most of the studies concentrated at the center of the funnel. However, there are more outliers on the left side as compared to the right side of the funnel. This shows there is a probability of publication bias in favor of tumor budding.

Discussion

This meta-analysis and systematic review examined the role of tumor budding in upper gastrointestinal carcinomas (UGIs) by combining information from 15 relevant studies with a total sample of 7607 individuals. According to the study findings, there is a statistically significant relationship between tumor budding and overall survival (P<0.00001). This was revealed with a mean difference of −0.09 at 95% CI (−0.20 to 0.02). According to the study by Li et al., shorter OS and PFS were found to have a statistically significant correlation with TB activity as determined by IHC staining per 20× objective lens (p < 0.05) [11]. However, in a study by Beer et al., there was no significant correlation found between the number of budding foci and survival in individuals with grade 3 tumors [12]. When DR was associated with TB in a study by Yavuz et al., it was revealed that DR is not the only functional independent predictor of survival; the study indicated that more investigation is necessary to fully understand its significance in predicting UGI behavior [13].

In regards to tumor differentiation status, the results show that there is a statistically significant association between tumor budding and tumor differentiation status (χ²=23.31, P<0.00001). The mean difference of the association was −12.60 at 95% CI (−35.89 to 10.68). However, in a study by Dhingra et al., one of the histopathologic characteristics linked to unfavorable results was inadequate differentiation [14]. In a univariate analysis in a study by Yu, a statistically significant relationship between LNM and tumor differentiation was noted. In addition, multivariate logistic regression analysis of the study revealed that tumor differentiation is one of the other independent risk factors for LNM [15]. According to multivariate logistic analysis in a study by Wang et al., tumor differentiation and LNM exhibited a strong correlation indicating that it is one of the major risk factors [16].

Depth of the tumor stage also had a statistically significant association with tumor budding (χ²=480472.97, P<0.00001), MD=−6.18 at 95% CI (−14.66 to 2.30). According to the finding in a study by Landau et al., in cases of superficial esophageal adenocarcinoma, significant tumor budding was noted as a risk factor for lymph node metastasis and was linked to a 2.5-fold increase in the likelihood of nodal metastasis [17]. Davison et al. observed that beyond the standard nodal staging system, the pathological features of primary superficial EACs could help identify patients with a low risk of metastasis, who may be suitable for endoscopic resection, as well as those at higher risk who might benefit from induction or adjuvant therapy [18]. Similarly, Dao et al. found that the five-year disease-free survival (DFS) rate was 88.2% in the low Bd group, while it was 95.0% for Bd1 and 84.7% for Bd2, highlighting differences across the stages [19].

Moreover, a statistically significant correlation was found between the lymph vascular invasion and tumor budding (χ²=29.59, P<0.00001), MD=−5.03 at 95% CI (−11.26 to 1.21). In a study by Kemi et al., it was noted that in cases of gastric adenocarcinoma, high tumor budding is an independent prognostic marker. However, for diffuse types, it is not predictive [20]. Teramoto et al.'s univariate analysis of vascular invasion and tumor budding revealed that the pathogenic variables significantly impacted the prognosis. In a multivariate analysis involving the pathologic characteristics as covariates, vascular invasion and lymphatic vessel invasion were found to be significant independent prognostic predictors [21]. Further, it was found in a study by Baydas et al. that the mean time from diagnosis to the last follow-up was significantly longer in patients with lymph vascular invasion [22].

There was a significant association between the lymph node metastasis and tumor budding (χ²=158.30, P<0.00001, with a mean difference of −3.44 at 95% CI (−4.72, −1.78). As noted in a study by Lee et al., when treating early gastric cancer (EGC), endoscopic resection (ER) is a minimally invasive therapeutic option that is especially useful for patients who have a low risk of lymph node metastasis (LNM) [23]. The study by Heckl et al. found a statistically significance and frequent expression of VIR and CC-IR in GC [24]. Finally, but not least, a study by Pun et al. found tumor budding and desmoplastic reaction to have a strong correlation with RFS (P=0.020) but not with OS (P=0.066) after removing four patients with metastatic lymph nodes. On the other hand, tumor budding was significantly correlated with OS and RFS in patients without metastatic lymph nodes when categorized using a two-tier grading system [25].

Some limitations exist with our meta-analysis. First, although patients were categorized into groups according to high-grade or low-grade tumor budding, the stratification may be inconsistent due to differing cut-off values across the included studies. The studies used varying thresholds for defining high-grade TB, while others employed different magnifications for identifying budding foci, leading to potential inconsistencies. Additionally, the inclusion of studies with diverse sample sizes may have influenced the overall estimates, with larger studies carrying more statistical weight. This heterogeneity suggests that the results should be interpreted with caution, as methodological differences may affect the reliability and generalizability of the findings. Future studies should aim to standardize TB assessment methodologies to reduce variability and improve comparability across research findings.

Second, we extracted a subset of IHC and its corresponding 95% confidence intervals from the survival curves. However, these numbers may not be as accurate as those directly derived from survival data. Third, because papers published in other languages were not included in the meta-analysis, there are chances of bias. Lastly, the included papers had relatively small sample sizes, which may have introduced biases. On that basis, future studies should focus on randomized control trial papers involving a large sample size without limitation on the publication language to give curated findings that can be applied in a wide context.

## Conclusions

The study reveals that there was a significant relationship between tumor budding and various prognostic factors, including overall survival, lymph node metastasis, tumor differentiation, and lymphovascular invasion in upper gastrointestinal carcinomas. Patients with UGI who had high-grade tumor budding also had adverse clinic pathological characteristics and an overall survival of approximately five years. Further, the study noted that tumor budding may be a distinct prognostic marker. In order to validate these findings, the study strongly recommends conducting additional research on larger preoperative UGI biopsies.
